# Miscellaneous Cases

**Published:** 1854-10

**Authors:** John C. Darby

**Affiliations:** Lexington, Ky.


### The Western Journal

OF

### MEDICINE AND SURGERY.

OCTOBER, 1854. NEW SERIES. VOL. II.—NO. 10.

### ART. I.—MISCELLANEOUS CASES.

BY JOHN C. DARBY, M. D., OF LEXINGTON, KY.

In the *Medical Examiner and Record of Medical Science*, for July, 1852, I find a notice of the following case in the *London Lancet:* "*London Hospital.*—Abscess of the Lung following Pneumonia; death; autopsy; under the care of Dr. PEREIRA;" with these remarks:—"The formation of abscess in the lung as a consequence of pneumonia is considered a very rare occurrence, but it is probably still more rare to find a circumscribed accumulation of pus in the pulmonary tissue taking on a semi-gangrenous character." Having recently had such a case, and as it seems to be so rare, I report it for the *Western Journal of Medicine.*

On the 17th of September, 1851, I was called to see Mr. JAMES R. BOLTON, blacksmith, some 23 or 24 years of age. His disease was typhoid fever of a very grave form. He was at the house of two poor widows, who had kindly taken him in when he was taken sick, and who informed me that he had been sick for near a week, and had been out of his head from the first. He had had diarrhœa from the first, and would several times during the day dress himself, say he was well and was going down town. He would go down stairs, and by the time he got there he was unable to go any farther. I found him in this condition, with the same disposition to go out of doors.

It is unnecessary to state the treatment for the typhoid fever. I had him removed to the house of a friend who was better able to attend to him, and in about three weeks he was convalescent. He seemed to be doing well for near a week, when he was suddenly attacked with pneumonia of the right lung. For the pneumonia I gave him the following mixture, (which by the way I recommend as a remedy that will suit as many cases of pneumonia after the first onset as any other formulary I have ever tried)—viz:

| | |
|---|---|
| Brandy and water, made of usual strength, | ℥viii. |
| Tartar. antimony, | grs.v. |
| Laudanum | ʒ. mix. |

The dose of this mixture is ℥ss. The quantity of tartar emetic may be increased or lessened. p. r. n.

He took a dose of this mixture every two hours, if not asleep. The pneumonia commenced about the 4th of October, by the 11th or 12th that disease seemed to be subdued, except that there was still an occasional pain in the side. He continued to improve for four or five days longer, when there commenced a very fœtid odor whenever he coughed. It smelled like suphuretted hydrogen, and the expectoration was purulent, and copious. I then decided that it had become a case of abscess of the lung. There was dullness on percussion over the right side, and the respiratory murmur was very indistinct. All that I did after this was to endeavor to sustain his strength. I gave nutritious food, brandy and cod-liver oil. He died on the night of the 25th of October. From the time that this fœtid odor first commenced up to the hour of his death, it continued, and his nurses were frequently compelled to leave the room on account of it. He was very much emaciated. His appetite had continued to be quite good from the time that he seemed to be well of typhoid fever. I considered him to be a young man of ordinarily good constitution.

*Autopsy.*—Drs. B. P. DRAKE, J. M. BRUCE, and W. CROMWELL assisted in the post mortem examination. There was nothing remarkable except in the right lung, in the central portion of which there was a circumscribed abscess containing near a pint of fœtid pus. The abscess had opened and discharged into the cavity of the chest. There was a large amount of pus about the lung on the right side.

*Remarks.*—My opinion is that the young man would have recovered from the typhoid fever and got well, had he not been seized with pneumonia; and that the latter degenerated into pulmonary abscess in consequence of the enfeebled state of his system consequent upon his protracted and severe illness.

---

Case 2.—Pneumonia of the left lung with solidification of that organ.

I was called to see Mr. N. B. E——. on Sunday evening, May 13, 1849. He had been treated for pneumonia for near a week, by two of the best physicians of the city. Before going to see the patient (who was a pauper case) I called on one of them, who informed me that I was at perfect liberty to take charge of the case, and that he regretted that he did not tell the family in the morning that he should not return, as he considered the case hopeless, that the left lung was completely solidified, &c., &c. I found the left lung to be as stated by Dr. ——. It was perfectly dull on percussion, and entirely impervious to the air. He also had had hiccups for twenty-four hours or longer, off and on, and it was very severe when I saw him. The pulse, though not very strong or full, yet as I thought, justified bleeding. The patient's voice was pretty good, and he seemed to have no idea of dying. His bowels were constipated. I bled him fully a quart, with very great relief. I then gave him twenty grains of calomel, and two hours afterwards gave him ten grains of musk. The musk was repeated every four or six hours according to the severity of the hiccups. By the next morning he was much better, but the hiccup continued for three days. During this time I gave twenty grains of calomel night and morning, and the musk as above. The patient also had good rich broths, and brandy milk punch. After the hiccup subsided and he began to rally I used tartar emetic in small quantities mixed with brandy and water, and laudanum as directed in the first case. I dismissed this patient on the 12th of June. But in the interval he had several returns of the hiccup and had several times appeared to be on the verge of the grave. He so far recovered his health as to give his usual weight, and to look as well as ever. He was able to attend to his business, and did well until the summer of 1851, when he began to be troubled with a bad cough. This turned out to be a rapid consumption, and he died early in October, 1851.

Accidentally, I was prevented from making a post mortem examination, which I very much regretted. I oftentimes examined this man after his first convalescence, but could never hear any respiratory murmur in the left lung, except high up near the clavicle.

*Remarks.*—Physicians should not despair too soon of a case of pneumonia when one lung is sound. Calomel in decided doses is one of the most valuable remedies in this disease when the bowels are not too much acted on; and when they are if you will give tartar emetic in combination with brandy and laudanum, as above directed, you may still give the calomel with great advantage- Of all the remedies for pneumonia, judiciously used, bleeding has perhaps saved as many lives in desperate cases as any other remedy. My opinion is that the bleeding, calomel and musk saved this man's life in the first instance, and that good treatment afterwards restored him to health and prolonged his life.

---

Case 3.—Obstetrics.

I was called to see Mrs. I——., aged twenty-eight years, on the night of July 5, 1852; there were slight labor pains—presentation natural. The patient continued in this condition until the morning of the 7th, when the pains became more severe. Nothing unusual occurred, and the lady was delivered of a fine hearty boy weighing about seven pounds, a few minutes after 5 o'clock in the afternoon. In removing the placenta I observed that there was a separate body of small size attached to it. When the placenta had all come away I examined this body, and easily tore it open with my fingers. I found within a fœtus about six inches long, perfectly developed as to the limbs and body, the head was about the size of a Spanish dollar and not much thicker, *i. e.* it was flat. I gave the dead fœtus to Prof. Letcher of Transylvania Medical College to show to the class. There was nothing unusual in this lady's case. She had been married several years. She says that she thinks that constant sewing was the cause of the death of the second child. The mother and son are, to-day, (July 15) doing very well.

In the Louisville *Daily Democrat*, for the 15th of Sept., I find a notice of "a Boston broker, Mr. George H. Goodin," having cut the tendo-Achilles with an adze, with this remark: "The physician states that the severed cords will never unite, and he must submit to be a cripple for life." This is a mere newspaper notice, and may not be entitled to any attention; but as I have treated a far worse case successfully, I send you a short notice of it for your medical journal.

Case 4.—On the 10th of October, 1852, I was called to see John Ackman, who had been employed to pull down a brick wall. Finding that a part of the wall was about to fall he turned in haste to make his escape, when the leg of his pantaloons at the ankle was caught by a nail and held him, and, as he says, about three hundred bricks fell on his leg. Both bones were broken about two and a half or three inches above the ankle. The tendo-achilles, for its entire length, was separated into strings not larger than a fiddle-string. The broken bones were adjusted, and a bandage applied from the ends of the toes to near the knee. Cold water dressings were then constantly applied, off and on, as necessary, until the patient recovered. The plan was to wrap carefully and gently over the bandage and around the wounded parts soft cloths wrung out of cold water, and then to put a dry cloth over the wet one, and to renew the wet cloth as often as it got too hot or began to dry. The broken bones united without difficulty, but the tendo-achilles sloughed for three or four inches, nevertheless a new cartilage was formed and on the 25th of December following I dismissed him cured. The parts were tender for some time, but he soon went to work as a day laborer and has ever since done the heaviest work, as lifting bags in a mill, &c., &c. To see him walk, no one would ever suspect that he had ever been injured, and to examine the leg you could hardly believe that the tendo-achilles was ever destroyed.

This case proves two things:—1st. The wonderful recuperative powers of nature. 2d. The great value of the bandage when properly applied; and 3d., I may add, the great virtue of cold water dressings.

*Sept.* 1854.

